# Social Group Differences in Influencing Factors for Chinese Urban Residents’ Subjective Well-Being: From the Perspective of Social Stratification

**DOI:** 10.3390/ijerph19159409

**Published:** 2022-07-31

**Authors:** Ping Wen, Jiting Zhang, Suhong Zhou

**Affiliations:** 1 School of Geography and Planning, Sun Yat-sen University, Guangzhou 510275, China; wenp@mail.sysu.edu.cn; 2 Zhuhai Institute of Urban Planning and Design, Zhuhai 519072, China; zhangjit@mail2.sysu.edu.cn

**Keywords:** social stratification, subjective well-being, influencing factors, neighborhood environment

## Abstract

With the great pressure of modern social life, the problem of residents’ subjective well-being has attracted scholars’ attention. Against the background of institutional transformation, China has a special social stratification structure. The socio-economic resources and living needs of different social classes are different, resulting in differences in the level of subjective well-being and the influencing factors for this. Taking Guangzhou as an example, based on the data of a household survey conducted in 2016, this paper obtains the social hierarchical structure through two-step clustering, and explores the differences between influencing factors for subjective well-being using multiple linear regression models. The clustering results divided Guangzhou urban residents into four classes: retirees, white-collar workers outside the system, manual workers and white-collar workers inside the system. The subjective well-being of white-collar workers inside the system and manual workers is high. The subjective well-being of white-collar workers outside the system is below the average value, and retirees have poor subjective well-being. The results of the regression analysis show that the subjective well-being of all social classes could be improved by active participation in fitness exercises, harmonious neighborhood relationships and a central residential location. Health-related factors such as physical health, sleeping time and density of neighborhood medical facilities, have a significant impact on manual workers’ subjective well-being. An increase in the density of neighborhood leisure facilities could help to improve the subjective well-being of white-collar workers outside the system. However, this would inhibit the subjective well-being of white-collar workers within the system. By revealing the differences in influencing factors for different social groups’ subjective well-being, the research conclusions could provide a reference for the formulation of targeted policies and measures to improve residents’ subjective well-being in urban China.

## 1. Introduction

For more than 40 years, with the implementation of economic system reform and opening-up policies, China’s economy has rapidly developed. After making great achievements, the development speed has slowed down and entered a new stage of transformation and adjustment. Before reform, the urban social space was organized in the form of *Danwei* (state-owned institutions), the distribution of housing, food and other materials was relatively homogeneous [1], and there was little difference in residents’ socio-economic status. After the reform, market forces were introduced and a series of changes were implemented in the *Danwei* system, housing policy, medical care policy and elderly care policy. The widening income gap has exacerbated social differentiation. At the same time, the inertia of the old *Danwei* system still plays a role. For example, the *Danwei* community still exists, and there are great differences in welfare between groups employed inside and outside the *Danwei* system, as well as between urban and rural groups [2]. The interaction between the old and the new forces has reconstructed China’s urban social structure and formed a social stratification situation with Chinese characteristics. The differences between social groups and the accelerated pace of life brought on by the rapid economic development have led to there being a great psychological pressure on residents, which may have a negative impact on subjective well-being. After the slowdown of economic development, the problem of residents’ subjective well-being that accumulated in the period of rapid development has increasingly attracted the attention of scholars in various fields. In recent years, the research focus of Chinese health geography has shifted from the geographical distribution of diseases to the problems of subjective well-being, geographical environment, social spatial differentiation and social equity [3,4,5,6,7]

In relevant studies, subjective well-being is defined as people’s overall feeling of happiness in life, or life satisfaction, which is generally measured by a single or multiple self-report scales [8]. The influencing factors of subjective well-being are mainly analyzed from the perspectives of geographical environment and individual attributes [9]. The influence of the geographical environment comes from the physical built environment [10] and social environment [3,5]. The residential environment is people’s main physical environment in their life. Improvements in the accessibility of medical, educational, leisure and other facilities can help to improve residents’ subjective well-being [11]. Transportation convenience and community greening are also positive factors affecting subjective well-being [11,12,13]. The improvement in housing conditions is conducive to improving residents’ mental health [14,15,16]. The neighborhood soft environment includes residents’ integration with and sense of belonging to the neighborhood. Neighborhood harmony means that residents have rich social support, a stronger adapting ability and psychological tolerance to the stressful social and cultural environment, and a weaker sense of deprivation caused by injustice, which could eliminate the negative impact of adverse events and improve individuals’ mental health and life satisfaction [17,18,19]. In terms of personal attributes, better economic conditions and a stable life are conducive to the improvement in residents’ subjective well-being. Poor living habits such as lack of sleep and lack of exercise are not conducive to mental health [20,21]. Physical health is highly related to subjective well-being [22]. In addition, due to the differences in socio-economic resources and the needs of different classes of people, as well as their different ability to adapt to the environment, socio-economic status has become an important factor determining individual subjective well-being. Relevant studies generally regard the level of socio-economic status as an influencing factor for individual subjective well-being and suggest that individuals with a high socio-economic status are more likely to have strong subjective well-being [23].

Recent empirical evidence from China has confirmed the impact of the geographical environment and individual characteristics on subjective well-being [24,25,26,27,28]. Furthermore, Chinese geographers have studied the subjective well-being and influencing factors for this in special groups such as the elderly [29], internal migrants [30], elderly migrants [31] and low-income classes [32]. The differences in subjective well-being between residents with different socioeconomic statuses and different geographical locations have been compared [33,34,35]. In the context of social stratification in China’s institutional transformation, the social and economic status of residents is difficult to simply characterize using any single variable, but should be comprehensively considered in terms of income, occupation, housing, education, registered residence and other factors [36,37]. While the subjective well-being of residents with different socio-economic statuses varies, the influencing factors of subjective well-being may also differ [34,35].

In summary, China’s institutional transformation and rapid economic development have exacerbated social stratification and social pressure. Under the new normal of economic and social development, residents’ subjective well-being has been a topic of concern for scholars. Existing studies believe that residents’ subjective well-being is affected by various factors such as geographical environment and personal attributes. There are significant differences in subjective well-being among groups with a different socio-economic status, but there is little research and few conclusions regarding the differences in influencing factors of subjective well-being among different social groups. This study believes that different social classes have different resources, different environments, different needs and different degrees of satisfaction of their needs, and their subjective well-being and influencing factors may be different. This paper will focus on the social stratum differences in influencing factors for urban residents’ subjective well-being against the background of social stratification in contemporary China. In this context, two research questions will be investigated in this paper: (1) What are the differences in subjective well-being among different social classes in urban China? (2) What are the differences in the influencing factors of subjective well-being among different social classes in urban China?

## 2. Data Sources and Research Methods

### 2.1. Study Area and Data Source

The southeast coastal area is the frontier for China’s reforms and opening-up. Industrialization and urbanization have developed rapidly since the 1980s, making it one of the most urbanized and developed regions in China. As a traditional core city in this area, Guangzhou has experienced profound institutional reform. The urban economy and society have developed comprehensively, playing a leading role as a national central city and a gateway city. The population of Guangzhou has increased from 4.82 million at the beginning of reform and opening-up in 1978 to 18.81 million at the end of 2021 [38]. The social strata within the city were fully developed after reform and opening-up. Only 8.51% of employed persons were employed by state-owned or urban collective-owned *Danwei* at the end of 2020, and the wages of employed persons vary between institutions and different economic sectors [39]. Therefore, this paper selected Guangzhou as the study area.

**Figure 1:**
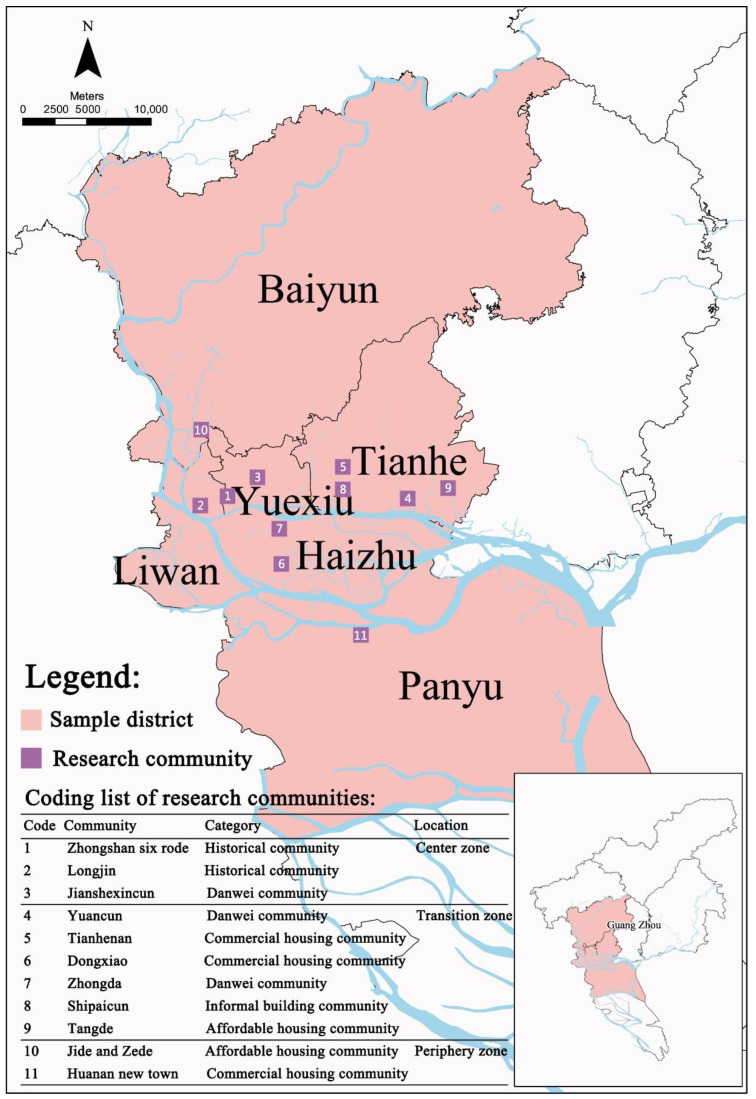
Distribution of research communities.

The data used in this study mainly come from a questionnaire survey of urban residents, carried out in Guangzhou in January 2016. The survey was conducted in the old urban area within the inner ring road of Guangzhou, the transition area between the inner ring road and the ring expressway, and the peripheral area outside the ring expressway. In the process of urban expansion, new forms of spatial blocks, such as market-oriented commercial housing communities, government-led affordable social housing communities, and bottom-up informal housing communities, were emerging and forming new built-up areas. To cover different people, 11 representative blocks in five typical communities [40,41,42], including historical communities, *Danwei* communities, commercial housing communities, social housing communities and informal housing communities, were selected for the survey. The distribution and community types of the investigated blocks are shown in Figure 1. The historical communities are located in the traditional urban center of Guangzhou, with a complex population composition that mainly includes traditional local residents and migrant workers who rent houses. *Danwei* communities are the legacy of the planned economy period. In these communities, housing is allocated to employees by *Danwei*, and the residents are mainly old employees of state-owned enterprises. Commercial housing communities are newly built housing after housing system reform. Residents obtain housing property rights through housing market purchases. Social housing communities offer affordable housing, provided by the government, to low-income residents. Informal housing communities are mainly self-built houses by villagers in urban villages on collective land, which are provided for villagers to live by themselves and migrant workers to rent.

Households were randomly entered from the selected blocks. One non-student adult from each household was invited to fill in the questionnaire face-to-face. As young students have not yet entered the workforce, and their social status is not yet clear and stable, they were not interviewed as respondents for this survey. The questionnaire mainly includes the following contents: personal characteristics, residence and employment, physical exercises, health and medical care, community participation and satisfaction. The questionnaire took about 20–30 min to complete the questionnaire. The participants provided their informed consent to participate in the survey. A total of 1029 valid questionnaires were collected. The male-to-female ratio of the sample was 49.95:50.05. The average age was 41.05 years old. A total of 78.04% of the samples were married; 71.04% had an average monthly income between 3000 and 6999 CNY; 77.74% were registered as local residence in the city.

### 2.2. Research Methods and Index System

This paper used the two-step clustering method in SPSS software to divide the social classes of urban residents. The two-step clustering was completed in two stages. In the first step of pre-clustering, all respondents were individually inserted into a Cluster Feature Tree (CF Tree), while outliers were removed and the CF Tree was rebuilt. Similar respondents were grouped and many sub-clusters were generated. The second step is clustering, in which the agglomerative hierarchical clustering method was used to merge the sub-clusters from the first step, until the most appropriate number of clusters was found based on Bayesian information criterion (BIC). Finally, all respondents were assigned to a cluster. Two-step clustering can simultaneously provide discrete variables and continuous variables for the clustering, and automatically judge the most suitable number of categories. This study selected six variables, namely income, the nature of the institution, occupation, nature of housing, education and residence registration, as the basis for class division [37,43]. Income represents the acquisition and possession of social wealth. The nature of the institution and the type of occupation determine the power status of the residents, to determine the degree of social security that they enjoy from the government. The nature of housing reflects the level of institutional security that residents’ enjoy. Their educational background determines residents’ ability to obtain social and economic resources. Residence registration represents the urban/rural and local/outsider status of residents registered by the government, which might be inconsistent with their actual residence and affect the level of welfare that one can enjoy, with local–urban registration usually meaning better welfare.

Subjective well-being is measured by the five-item WHO well-being index [44], which comprises five items: ‘I have felt cheerful and in good spirits’, ‘I have felt calm and relaxed’, ‘I have felt active and vigorous’, ‘I woke up feeling fresh and rested’, and ‘My daily life has been filled with things that interest me’. A six-point Likert scale, where 0 stands for ‘at no time’ and 5 for ‘at all time’, is used to score the respondents’ feelings regarding the above five aspects during the last two weeks. The total score ranges from 0 to 25, with a higher score meaning better subjective well-being. In this study, the total score for the samples’ subjective well-being ranged from 2 to 25. The average value was 12.09, and the standard deviation was 3.692, which is an approximately normal distribution.

Personal demographic characteristics, health-related characteristics and neighborhood environmental characteristics are tested as influencing factors for each social groups’ subjective well-being. Individual attributes include three basic characteristics: gender, age and marriage. The six socio-economic variables which were used to divide the social groups into two-step clustering were not included again, Physical health, daily sleep time and weekly fitness time were used to measure residents’ health and lifestyle. Physical health was characterized by the respondents’ answer to “have you suffered from physical pain in the past month”. ‘All the time’ was recorded as 1, while ‘at no time’ was recorded as 5. The higher the score, the healthier the body. The neighborhood environment variables mainly investigate three major items: residential location, neighborhood facility densities and neighborhood relationship. Residential location was measured by the distance from the residence to the population center. Neighborhood facility densities were characterized by the points of interest (POI) densities of various facilities in the 500-m buffer zone of the residence. According to the existing research, four types of facilities were investigated: retailing, education, medicine and leisure. Neighborhood relationship is characterized by the respondents’ answer to five questions, namely, ‘in our neighborhood, people are willing to help each other’, ‘neighbors often visit each other’, ‘people in the neighborhood are trustworthy’, ‘people in the neighborhood say hi to each other when they meet’ and ‘if there is a problem in the neighborhood, people can get together to deal with it’. The total score ranged from 5 to 25. The higher the score, the more harmonious the neighborhood relationship.

The linear regression model of least squares estimation in SPSS software was used to test the influencing factors of subjective well-being for each social group. The collinearity of the independent variables was diagnosed in the regression process.

## 3. Social Stratification and Group Differences in Influencing Factors of Subjective Well-Being

### 3.1. Social Stratification of Urban Residents in Guangzhou

Based on six social-economic variables, the two-step clustering method was used to cluster all 1029 samples. The samples were divided into four categories, and the cluster quality was “good”. Among the six variables, the predictive importance of occupation type was the highest (1.00), followed by the nature of the institution (0.56), education (0.30) and income (0.20), and the importance of the nature of housing (0.05) and residence registration (0.01) was the lowest. The sample sizes for the four clusters were 163, 389, 266 and 211, respectively.

The social-economic characteristics of the four clusters are shown in Table 1. Samples in the first cluster are mainly unemployed people with a lower educational level and income. Further investigation shows that their average age was 62.13, and those aged 50 and above accounted for 88.96%. Therefore, this cluster was named retirees. Samples from the second cluster are mainly white-collar workers employed by non-state owned institutions and self-employed persons. Their income and educational levels were relatively higher, and a small number of them had a high income. They are named white-collar workers outside the system. Samples in the third cluster are generally manual workers such as factory workers and waiters, with relatively low education and income levels. Nearly 80% of them were employed by non-state-owned units. They were named manual workers. Samples in the fourth cluster are mainly staff employed in state-owned institutions, with relatively higher educational and income levels, and a high proportion of them enjoyed government-subsided housing. This group was named white-collar workers inside the system.

### 3.2. Subjective Well-Being and Characteristics of Different Social Classes in Guangzhou

The subjective well-being, personal attributes and neighborhood environmental characteristics of the samples of four social groups are shown in Table 2. Due to the lack of variable values in some samples, the four types of samples included in this round of analysis were 132, 363, 244 and 203, respectively, accounting for 91.55% of the total samples.

The average score of subjective well-being for white-collar workers inside the system is the highest among the four groups, followed by that of manual workers. The subjective well-being of white-collar workers outside the system is lower than the average value of all samples, and that of the retirees is the lowest. Manual workers and white-collar workers outside the system have the best physical health, followed by white-collar workers within the system, and retirees have the worst health status. Retirees spend the most time on exercise and fitness every week, while employees, especially white-collar workers, spend less time on exercise. In terms of neighborhood environment, the centrality of retirees’ residence is the highest, and they also enjoy high POI densities in various service facilities. White-collar workers inside the system and manual workers live near the center of city, and the density of their neighborhood facilities is high. White-collar workers outside the system live far away from the urban center, and the density of their neighborhood facilities is significantly lower than that of the other three groups. The neighborhood relationship of manual workers is the most harmonious, followed by retirees and white-collar workers within the system, and the neighborhood relationship of white-collar workers outside the system is the worst.

Overall, although white-collar workers inside the system have poorer physical health, they have a higher socio-economic status, better living environment, and the most adequate welfare protection, and generally have higher scores of subjective well-being. The social and economic status of manual workers is relatively low, but they have healthy bodies, a more vibrant neighborhood environment, and better subjective well-being. White-collar workers outside the system experience greater living pressures, have limited participation in leisure activities such as fitness and exercise, live in the suburbs with insufficient service facilities, rarely have close neighborhood relationships, and have low subjective well-being. Retirees’ health is relatively poor, and their subjective well-being is low.

### 3.3. Social Group Differences in Influencing Factors for Subjective Well-Being

Multiple linear regression models were used to analyze the influencing factors for different social classes’ subjective well-being. The results are shown in Table 3.

The influencing factors for the subjective well-being of different social groups have similarities and differences. In terms of commonness, the weekly fitness time has a positive impact on the subjective well-being of samples from all groups. The regression coefficients and significance level of weekly fitness time for the white-collar classes both inside and outside the system are higher, indicating that the positive effect of increasing fitness level is clear. The residential centrality level can help to improve the subjective well-being of all classes. The degree to which this improves subjective well-being is highest for retirees, indicating that retirees have the highest preference for a central urban area. In addition, harmonious neighborhood relations also help to improve the subjective well-being of all social classes.

In terms of differences in influencing factors, health-related factors have particularly positive effects on the subjective well-being of manual workers. In addition to the above-mentioned weekly fitness time, increases in daily sleep time and neighborhood medical facility density, as well as the enhancement of physical health, could significantly improve manual workers’ subjective well-being. The density of neighborhood leisure facilities has the opposite effect on white-collar workers outside the system and inside the system. As white-collar workers outside the system have lower density of neighborhood leisure facilities, the enhancement of leisure facilities can significantly improve their subjective well-being. White-collar workers inside the system enjoy a high density of leisure facilities. Further improvements in the density will have a negative effect on subjective well-being, which may be due to the noisy neighborhood environment caused by leisure and the entertainment activities attracted by leisure facilities. In addition, there is an inverted U-shaped relationship between age and the subjective well-being of white-collar workers outside the system. The subjective well-being of white-collar workers outside the system reaches a relatively high level around the age of 36, and then their subjective well-being decreases with age.

## 4. Conclusions

Based on the questionnaire survey data from urban residents in Guangzhou in 2016, this study found that there were not only differences in subjective well-being among different social groups, but also differences in the factors influencing this. The two-step clustering method was used to divide the samples into four social classes—retirees, white-collar workers outside the system, manual workers and white-collar workers inside the system—by incorporating six variables of income, the nature of the institution, occupation, the nature of housing, education and residence registration. Among the four classes, white-collar workers inside the system have the highest levels of subjective well-being. The subjective well-being of manual workers is slightly lower. White-collar workers outside the system have a lower level of subjective well-being than average in the total sample. Retirees’ subjective well-being is the worst. The multiple linear regression results show that health-related factors such as physical health, daily sleep time and the POI density of neighborhood medical facilities have a positive impact on the subjective well-being of manual workers. The neighborhood leisure facilities’ POI density has a positive impact on the subjective well-being of white-collar workers outside the system who live in the suburbs with a lower density of neighborhood facilities. However, for white-collar workers inside the system, the increase in the density of neighborhood leisure facilities’ POI reduces their subjective well-being. Improvements in weekly fitness time, residential location centrality and neighborhood relationship can promote the subjective well-being of all classes.

The results of the social class division in this study show that, against the background of institutional transformation, institutional factors are still key elements of social stratification of urban residents in China. Different social classes from inside and outside the state-related system have different employment and income stability, different socio-economic resources, different needs and different demands for their satisfaction, so their subjective well-being and the related influencing factors are also different [45].

Employees inside the system have stable employment, high income levels, and perfect welfare security, and are in a relatively high position in terms of social stratification, with good subjective well-being. Besides the general influencing factors, they have no special needs to be met.

White-collar workers outside the system represent an emerging middle-income group in the process of institutional transformation. The development and growth of this group is of great significance for the formation of an olive-shaped modern social structure [46]. This class seems to have decent work and relatively high income, but they face greater market risks. They have higher demands for welfare security, which cannot be fully satisfied in reality [47], and their subjective well-being is at a low level. People from this class are often buyers of commercial housing in the newly built suburbs, where the quantity and quality of service facilities are lower than those in the central urban area. Their living pressure is further increased by the burden of long-distance commuting Improvements of the leisure and other facilities could help to improve their subjective well-being.

Manual workers have lower levels of personal qualities such as education, but usually have more conservative requirements and expectations for life. They are typically more easily contented. Therefore, although they are in a relatively low position in terms of overall social stratification, their subjective well-being is at a relatively high level, only slightly lower than that of white-collar workers inside the system. As the most important element of human capital, having a healthy physique has the strongest impact on manual workers’ subjective well-being.

Most retirees have dropped out of the labor market. The cumulative loss in physical health caused by decades of work and the natural aging of the body means that retirees are in generally poor health. Their low income level also reduces their ability to actively control their own lives. Therefore, retirees’ subjective well-being is low, and requires the attention of relevant policies [48,49].

Based on the results, this study attempts to put forward policy suggestions to improve the subjective well-being of different social classes, especially relatively vulnerable classes. Promoting the national fitness campaign and constructing harmonious communities will help to improve the subjective well-being of all social classes. As an emerging middle-income class, white-collar workers outside the system have benefited greatly from institutional reform and economic development, but it is difficult for them to improve their subjective well-being. In addition to strengthening and improving social security in the suburbs where they live, the quality of service facilities should be improved, and the quality of life in suburban communities should be comprehensively improved by better community governance. In view of the strong health needs of manual workers, medical security and medical facilities should be further improved. In the context of the aging society, the construction of an elderly-friendly neighborhood is also an important topic for urban planning and social construction.

There are still some limitations and deficiencies in this study. For a very large city with a permanent population of nearly 20 million, the sample size for the survey in this study is relatively small. Although the diversity of the population and community types was considered in sampling, it is still difficult to fully reflect the richness of the urban population, so the resulting social class division is relatively rough. In addition, the variables collected based on the questionnaire and related data are relatively limited, so the influencing factors of subjective well-being considered in this paper are insufficient. Future research needs to further combine big data and small data, and research methods should be improved to enhance the reliability of the research results.

## Figures and Tables

**Table 1:** Social-economic characteristics of the four clusters.

	Retirees	White-Collar Workers Outside the System	Manual Workers	White-Collar Workers Inside the System
*Personal monthly income (CNY)*				
<1000	11.66	0.00	0.00	0.00
1000–1499	2.45	0.00	0.75	0.00
1500–2999	23.31	3.08	3.38	3.79
3000–4999	52.76	31.62	45.86	31.28
5000–6999	9.20	34.19	38.72	39.34
7000–8999	0.61	15.94	8.65	17.54
9000–12,000	0.00	3.34	2.63	5.69
>12,000	0.00	11.83	0.00	2.37
*Nature of institution*				
Public	0.00	0.26	19.92	98.58
Non-public	4.29	98.20	78.95	0.95
Not applicable	95.71	1.54	1.13	0.47
*Occupation*				
Officials in institutions	0.00	0.00	0.00	96.21
White collar workers	3.07	80.98	0.00	0.00
Manual workers	1.23	0.00	100.00	3.32
Self-employed	0.00	18.77	0.00	0.00
Not employed	95.71	0.26	0.00	0.47
*Nature of housing*				
Commercial housing	50.92	57.84	67.29	44.55
Affordable housing	3.07	7.97	6.02	15.17
Low-rent housing	1.23	2.83	1.13	1.42
Public rental housing	0.61	2.83	3.01	0.95
Staff dormitory	9.20	12.85	6.77	21.33
Bought public housing	9.20	9,25	8.27	9.00
Resettling housing	1.84	1.80	2.26	2.37
Self-built housing	23.93	4.63	5.26	5.21
*Education*				
Primary or junior school	68.10	4.37	8.27	3.32
Senior or technical school	30.67	77.63	75.19	66.35
College or above	1.23	17.99	16.54	30.33
*Residence registration*				
Local-urban	84.66	71.98	73.31	74.41
Local-rural	2.45	1.54	5.64	2.37
Migrant-urban	8.59	13.88	12.41	14.69
Migrant-rural	4.29	12.60	8.65	8.53
Number of cases	163	389	266	211

**Table 2:** Personal and neighborhood characteristics of the four social classes.

	Retirees	White-Collar Workers Outside the System	Manual Workers	White-Collar Workers Inside the System
Subjective well-being	11.16	11.85	12.49	12.76
*Personal characteristics*				
Gender: male (%)	43.18	50.41	51.23	49.26
Gender: female (%)	56.82	49.59	48.77	50.74
Age	62.15	35.75	37.84	37.31
Marriage: married (%)	97.73	73.83	72.95	75.37
Marriage: not married (%)	2.27	26.17	27.05	24.63
Physical health	3.95	4.47	4.48	4.19
Daily sleep time (h)	6.73	7.36	7.20	7.46
Weekly fitness time (h)	5.13	3.34	4.03	3.60
*Neighborhood environment*				
Residential location (km)	4.34	5.99	5.36	4.93
Retailing (POI/km^2^)	191.36	143.87	192.25	185.15
Education (POI/km^2^)	14.81	12.25	14.30	13.97
Medicine (POI/km^2^)	43.43	33.74	45.54	41.13
Leisure (POI/km^2^)	17.65	15.90	18.41	18.10
Neighborhood relationship	15.13	14.24	15.77	14.57
Number of cases	132	363	244	203

**Table 3:** Influencing factors of subjective well-being for different social classes.

	Retirees		White-Collar Workers Outside the System		Manual Workers		White-Collar Workers Inside the System	
	B	S.E.	B	S.E.	B	S.E.	B	S.E.
*Personal characteristics*								
Male (ref: female)	−1.442 ***	0.536	−0.284	0.317	0.367	0.414	0.475	0.450
Age	0.275	0.278	0.215 *	0.128	−0.196	0.154	−0.076	0.168
Age squared	−0.003	0.002	−0.003 *	0.001	0.002	0.002	0.001	0.002
Married (ref: not married)	−1.461	1.672	−0.628	0.505	−0.999	0.704	−0.752	0.702
Physical health	−0.246	0.335	0.151	0.254	0.748 **	0.352	0.005	0.344
Daily sleep time	0.205	0.275	−0.110	0.206	0.918 ***	0.225	−0.137	0.274
Weekly fitness time	0.118 *	0.062	0.186 ***	0.050	0.138 **	0.063	0.190 ***	0.060
*Neighborhood environment*								
Residential location	−0.507 ***	0.124	−0.322 ***	0.057	−0.172 **	0.067	−0.325 ***	0.079
Retailing POI density	0.000	0.001	0.000	0.001	0.001	0.001	−0.001	0.001
Educational POI density	0.005	0.055	0.008	0.034	0.025	0.037	0.016	0.049
Medical POI density	−0.004	0.018	0.010	0.011	0.026 *	0.015	0.014	0.015
Leisure POI density	−0.009	0.029	0.037 *	0.019	0.021	0.024	−0.050 *	0.027
Neighborhood relationship	0.348 ***	0.093	0.414 ***	0.053	0.474 ***	0.084	0.432 ***	0.090
Constant	2.658	8.249	3.162	3.258	−1.710	4.174	10.789 **	4.576
N	132		363		244		203	
Adjusted R square	0.318		0.389		0.317		0.287	

Note: B stands for coefficient. S.E. stands for standard error. *** *p* < 0.01; ** *p* < 0.05; * *p* < 0.1.

## Data Availability

The data are not publicly available due to privacy issues. Requests to the datasets should be directed to Suhong Zhou, eeszsh@mail.sysu.edu.cn.
